# Supplementary material for: Modeling glioblastoma heterogeneity as a dynamic network of cell states
Source: Mol Syst Biol. 2021 Sep 16;17(9):e10105. doi: 10.15252/msb.202010105 (PMC8444284; doi:10.15252/msb.202010105)
Supplement: Supplementary file 6 — Source Data for Figure 5 [file MSB-17-e10105-s004.zip › Figure5A_sourcedata/GSEA_3071/hallmarks_stateI.GseaPreranked.1623742531165/HALLMARK_FATTY_ACID_METABOLISM.html]

Details for gene set HALLMARK\_FATTY\_ACID\_METABOLISM[GSEA]

|  || Dataset | state13071 |
| Phenotype | NoPhenotypeAvailable |
| Upregulated in class | na\_neg |
| GeneSet | HALLMARK\_FATTY\_ACID\_METABOLISM |
| Enrichment Score (ES) | -0.22151248 |
| Normalized Enrichment Score (NES) | -0.87096286 |
| Nominal p-value | 0.62731874 |
| FDR q-value | 0.69659513 |
| FWER p-Value | 1.0 |
Table: GSEA Results Summary

  

Fig 1: Enrichment plot: HALLMARK\_FATTY\_ACID\_METABOLISM      
 Profile of the Running ES Score & Positions of GeneSet Members on the Rank Ordered List

  

| PROBE | GENE SYMBOL | GENE\_TITLE | RANK IN GENE LIST | RANK METRIC SCORE | RUNNING ES | CORE ENRICHMENT || 1 | OSTC |  |  | 151 | 0.915 | 0.0100 | No |
| 2 | UGDH |  |  | 238 | 0.759 | 0.0296 | No |
| 3 | MGLL |  |  | 406 | 0.581 | 0.0155 | No |
| 4 | EPHX1 |  |  | 429 | 0.570 | 0.0424 | No |
| 5 | ME1 |  |  | 543 | 0.484 | 0.0382 | No |
| 6 | GABARAPL1 |  |  | 544 | 0.484 | 0.0664 | No |
| 7 | CRYZ |  |  | 576 | 0.466 | 0.0847 | No |
| 8 | S100A10 |  |  | 673 | 0.422 | 0.0818 | No |
| 9 | HSD17B11 |  |  | 779 | 0.389 | 0.0743 | No |
| 10 | HMGCS1 |  |  | 994 | 0.328 | 0.0320 | No |
| 11 | SERINC1 |  |  | 1119 | 0.303 | 0.0140 | No |
| 12 | HADHB |  |  | 1271 | 0.274 | -0.0134 | No |
| 13 | SDHD |  |  | 1299 | 0.269 | -0.0055 | No |
| 14 | UBE2L6 |  |  | 1370 | 0.256 | -0.0106 | No |
| 15 | FH |  |  | 1552 | -0.262 | -0.0473 | No |
| 16 | ENO2 |  |  | 1707 | -0.281 | -0.0752 | No |
| 17 | ACAA1 |  |  | 1862 | -0.300 | -0.1020 | No |
| 18 | SUCLG2 |  |  | 1988 | -0.316 | -0.1194 | No |
| 19 | ALDH3A2 |  |  | 2040 | -0.324 | -0.1152 | No |
| 20 | HSP90AA1 |  |  | 2156 | -0.344 | -0.1282 | No |
| 21 | CCDC58 |  |  | 2161 | -0.345 | -0.1092 | No |
| 22 | KMT5A |  |  | 2228 | -0.360 | -0.1072 | No |
| 23 | ADSL |  |  | 2429 | -0.398 | -0.1414 | No |
| 24 | NCAPH2 |  |  | 2443 | -0.400 | -0.1218 | No |
| 25 | ECI1 |  |  | 2500 | -0.414 | -0.1137 | No |
| 26 | YWHAH |  |  | 2725 | -0.479 | -0.1501 | No |
| 27 | PCBD1 |  |  | 2759 | -0.490 | -0.1310 | No |
| 28 | CYP1A1 |  |  | 2886 | -0.533 | -0.1362 | No |
| 29 | G0S2 |  |  | 3184 | -0.762 | -0.1771 | Yes |
| 30 | ODC1 |  |  | 3257 | -0.860 | -0.1476 | Yes |
| 31 | PTPRG |  |  | 3308 | -0.945 | -0.1068 | Yes |
| 32 | GPD1 |  |  | 3506 | -2.825 | 0.0014 | Yes |
Table: GSEA details [plain text format]

  

Fig 2: HALLMARK\_FATTY\_ACID\_METABOLISM: Random ES distribution      
 Gene set null distribution of ES for **HALLMARK\_FATTY\_ACID\_METABOLISM**
